# EMNPD: a comprehensive endophytic microorganism natural products database for prompt the discovery of new bioactive substances

**DOI:** 10.1186/s13321-023-00779-9

**Published:** 2023-11-28

**Authors:** Hong-Quan Xu, Huan Xiao, Jin-Hui Bu, Yan-Feng Hong, Yu-Hong Liu, Zi-Yue Tao, Shu-Fan Ding, Yi-Tong Xia, E Wu, Zhen Yan, Wei Zhang, Gong-Xing Chen, Feng Zhu, Lin Tao

**Affiliations:** 1 Key Laboratory of Elemene Class Anti-cancer Chinese Medicines, School of Pharmacy, Hangzhou Normal University, Hangzhou, 311121 China; 2 College of Pharmaceutical Sciences, The Second Affiliated Hospital, Zhejiang University School of Medicine, Zhejiang University, Hangzhou, 310058 China; 3 Innovation Institute for Affiliated Intelligence in Medicine of Zhejiang University, Alibaba-Zhejiang University Joint Research Center of Future Digital Healthcare, Hangzhou, 330110 China; 4 Rehabilitation and Nursing School, Hangzhou Vocational & Technical College, Hangzhou, 310018 Zhejiang China; 5 The Affiliated Hospital of Hangzhou Normal University, Hangzhou, 310000 China; 6 First Clinical Medical Institute, Nanjing University of Chinese Medicine, Nanjing, 210023 Jiangsu China

**Keywords:** Endophyte, Natural product, Bioactivity, Natural product content, Microorganism natural product database, Drug discovery

## Abstract

The discovery and utilization of natural products derived from endophytic microorganisms have garnered significant attention in pharmaceutical research. While remarkable progress has been made in this field each year, the absence of dedicated open-access databases for endophytic microorganism natural products research is evident. To address the increasing demand for mining and sharing of data resources related to endophytic microorganism natural products, this study introduces EMNPD, a comprehensive endophytic microorganism natural products database comprising manually curated data. Currently, EMNPD offers 6632 natural products from 1017 endophytic microorganisms, targeting 1286 entities (including 94 proteins, 282 cell lines, and 910 species) with 91 diverse bioactivities. It encompasses the physico-chemical properties of natural products, ADMET information, quantitative activity data with their potency, natural products contents with diverse fermentation conditions, systematic taxonomy, and links to various well-established databases. EMNPD aims to function as an open-access knowledge repository for the study of endophytic microorganisms and their natural products, thereby facilitating drug discovery research and exploration of bioactive substances. The database can be accessed at http://emnpd.idrblab.cn/ without the need for registration, enabling researchers to freely download the data. EMNPD is expected to become a valuable resource in the field of endophytic microorganism natural products and contribute to future drug development endeavors.

## Introduction

Endophytes, a distinctive group of microbes residing within plants, establish a mutually beneficial relationship with their host plants [1]. They actively support their hosts in combatting biotic (e.g., pathogen infection) and abiotic (e.g., drought, extreme temperatures, salinity) stresses by producing a wide range of bioactive natural products (NPs) [2, 3]. NPs serve as the origin of modern pharmaceuticals [4], with approximately 49.5% of FDA-approved drugs over the past 40 years being NPs or their derivatives [5]. Considering the high demand and extraction limitations of NPs from plants [6, 7], microorganisms, especially endophytes, are regarded as promising sources of novel bioactive substances [8], including antibiotics and anticancer agents [9, 10]. With over 300,000 species of higher plants on Earth, each plant hosts one or more endophyte [11], the immense species diversity of endophytes and their potential for biosynthesis drive chemical research on these microorganisms [12, 13]. Endophytes are also known to possess the ability to produce metabolites similar to those of their host plants, exhibiting comparable bioactivity. Since the isolation of endophytic fungus producing paclitaxel from *Taxus brevifolia* by Stierle in 1993 [14], numerous NPs with potent bioactivities have been discovered [15–17], fueling global interest among researchers in exploring bioactive substances derived from endophytic microorganisms and positioning it as a leading area of innovation in drug development.

A significant wealth of biological activity data regarding endophyte NPs has already accumulated in the field. Constructing databases utilizing this valuable data is crucial for the advancement of microbial NPs. Currently, the landscape of NP structural databases is highly fragmented. Despite the availability of numerous NP databases, options specifically tailored for microbial NPs are surprisingly limited [18]. The currently largest open-access NPs database, COCONUT [19], contains 407,270 NPs, including 134,379 annotated with taxonomical origins [20], while NPASS 2.0 [21], CMNPD [22], and ChEMBL [23] provide highly detailed quantitative biological activity values data. However, these databases encompass a broader scope, lacking a specific focus on microorganisms and instead including plants, algae, and other species. Other large general compound databases like SuperNatural III [24] and ChemSpider [25] contain a vast amount of compound information, they lack species origins and comprehensive compound bioactivity data. In the realm of microbial NP databases, several notable resources that stand out as publicly accessible options, as shown in Table 1. These databases include MyxoDB [26], mVOC 3.0 [27], StreptomeDB 3.0 [28], and Natural Products Atlas 2.0 [29], which contain 674, 2061, 6524, and 33,372 NPs, respectively. Each microbial NP database offers its distinct focus, MyxoDB specializes in Myxobacterial NPs, mVOC focuses on microbial volatiles, StreptomeDB concentrates on Streptomycetes NPs, and Natural Products Atlas stands out as the largest open-access repository of microbial NPs, encompassing an astonishing number of compounds. Nevertheless, none of these databases provide quantitative biological activity values data for the compounds they house and comprehensive coverage specifically targeting endophytic microorganisms. Furthermore, it’s important to highlight that certain endophyte NPs mentioned in the literature have not been incorporated into these databases. Therefore, there is an urgent need for an endophytic microorganism NPs bioactivity database.

**Table 1:** A variety of open-access databases available for providing the data of microbial NPs (the first is the new database proposed in this study, while the remaining ones are sorted based on the number of NPs they contain)

Database	Description		URL	Number of NPs	Number of species source	NPs bioactivity	NPs content data	NPs quantitative biological activity values data
EMNPD		Endophyte NPs	1^a^	6632	1017	√	√	√
MyxoDB		Myxobacterial NPs	2^b^	674	√	×	×	×
mVOC		Microbial volatiles	3^c^	2061	1034	×	×	×
NPcVar		Plant, microbial NPs	4^d^	2201	694	×	√	×
StreptomeDB		Streptomycetes NPs	5^e^	6524	3302	√	×	×
CMNPD		Marine NPs	6^f^	31,561	3354	×	×	√
NPAtlas		Microbial NPs	7^g^	33,372	√	×	×	×
NPASS		Plant, microbial NPs	8^h^	96,481	32,287	×	√	√
COCONUT		Plant, microbial NPs	9^i^	406,747	60,171	×	×	×

The existence and non-existence of certain data type were indicated using ‘√’ and ‘×’, respectively. In the ‘Number of Species Source’ column, MyxoDB and NPAtlas did not provide specific statistics, so we used ‘√’ to indicate their presence

^a^http://emnpd.idrblab.cn/

^b^https://www.myxonpdb.sdu.edu.cn

^c^https://bioinformatics.charite.de/mvoc/

^d^http://npcvar.idrblab.net/

^e^http://www.pharmbioinf.uni-freiburg.de/streptomedb

^f^https://www.cmnpd.org/

^g^https://www.npatlas.org/

^h^https://bidd.group/NPASS/index.php

^i^https://coconut.naturalproducts.net/

In this study, we have developed a new database called EMNPD, the first comprehensive database describing endophytic microorganisms and their NPs bioactivity. EMNPD includes not only the systematic classification of endophytes as well as the geographic distribution of their host plants, but also the physico-chemical and ADMET properties information, quantitative activity data and their NPs contents data and their fermentation conditions. Most importantly, we have provided evaluative annotations of the biological activities’ potency of the NPs, ranging from high, moderate, and low activities to active or inactive. EMNPD aims to provide the scientific community with an open-access knowledge repository for studying endophytic microorganisms and their NPs, to help discover more valuable bioactive substances and promote research and development of new drugs. The database can be accessed at http://emnpd.idrblab.cn/, and all the data can be freely downloaded by users without registration.

## Construction and content

### Data extraction and curation

**Fig. 1:**
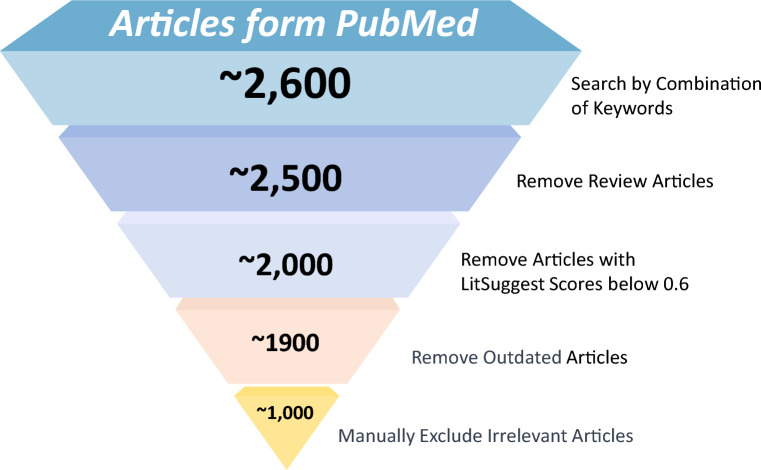
Workflow for literature filtering of EMNPD

The data in the EMNPD database was gathered from literature and various web repositories, following a series of sequential steps. Firstly, we utilized keyword combinations such as “endophytic microorganisms”, “endophytic fungi”, “endophytic bacteria”, “endophyte”, “natural product”, “compound”, “secondary metabolite”, “volatile”, “bioactivity”, “biological activity”, and “novel” to search for relevant articles in PubMed. This initial search yielded a total of 2600 articles. Secondly, we excluded review articles, resulting in 2500 remaining articles. Next, we utilized the LitSuggest [30], a web-based system that employs advanced machine learning techniques to predict and evaluate similarity scores among various articles, to filter out unrelated articles. To train the model, we assembled a positive dataset of around 100 manually selected literature sources closely associated with endophytic NPs. Additionally, a negative dataset was generated automatically and was twice the size of the positive dataset. Once the models were trained, they were employed to predict similarity scores for the aforementioned 2500 articles. After a thorough evaluation of these predictions, we identified articles with scores below 0.6 that did not align with our intended objectives. Consequently, we chose to exclude all articles that fell below this threshold. Furthermore, certain older articles that were challenging to access or lacked standardized data formats and essential information were omitted, ultimately leaving us with approximately 1900 articles for further analysis. Finally, through meticulous manual curation, we carefully selected and collected the content of 1000 articles. Figure 1 provides an overview of the literature filtering methodology used to construct the EMNPD.

### Data collection and processing

#### NPs data retrieval and characterization

**Fig. 2:**
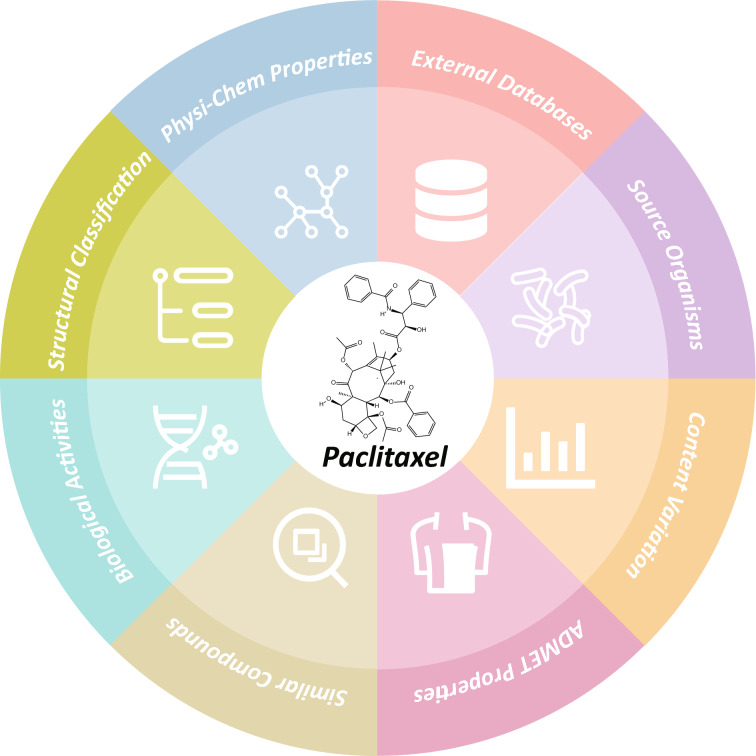
The knowledge graph of the individual compound information in EMNPD showcases a comprehensive range of data, using paclitaxel as an exemplary compound. It encompasses a diverse array of information, including physico-chemical properties, source organisms, NP content variations, biological activity, structural classification, similar NPs and drugs, external database links, and ADMET properties

All NPs data in the EMNPD were retrieved from various web repositories as well as a range of computational tools widely used in the field of chemical research. The compound names were extracted and collected from meticulously curated literature, as mentioned above. Based on these names, the Python package PubChemPy (version 1.04) and manual search were used to obtain the PubChem CID for the compounds. Based on these PubChem CID, their basic information, including 2D structure, molecular weight (MW), monoisotopic mass, calculated octanol-water partition coefficient (ALog *P*), topological polar surface area (TPSA), as well as the number of rotatable bonds (RB), number of hydrogen bond acceptors (HBA), number of hydrogen bond donors (HBD), aromatic rings (AR), and heavy atoms (HA), was collected from PubChem. For whole new compounds without a PubChem CID, their chemical structures were manually drawn based on the molecular images from the literature to ensure accurate representation. To enhance the efficiency of structure extraction, the optical chemical structure recognition tool KingDraw (http://kingdraw.cn/) was used to convert the graphic representation of the chemical structure into a machine-readable format. The converted structures were then manually reviewed, corrected, and double-checked using the chemical editor ChemDraw (version 20.0). The results were saved as mol and png files containing the structural information of the compounds. Subsequently, their basic information and physico-chemical properties were calculated using RDKit (https://www.rdkit.org), and their IUPAC names were predicted using STOUT [31]. All compounds were classified into the corresponding chemical categories using the ClassyFire web server [32]. ADMET properties were calculated using ADMETlab 2.0 [33], encompassing various parameters such as Caco-2 permeability, blood–brain barrier penetration, CYP1A2 inhibition, clearance, human hepatotoxicity, and more. Furthermore, using RDKit, we computed the similarity between compounds in EMNPD and each compound in the database, as well as all FDA-approved drugs in the TTD database [34], based on their SMILES, recording the top ten compounds and drugs with the highest similarity scores. A knowledge graph of the EMNPD compound is shown in Fig. 2.

#### Endophyte taxonomy and corresponding information

For all endophytic microorganisms in EMNPD, their Taxonomy ID and scientific names were obtained by searching the NCBI Taxonomy database [35] using their names extracted from the literature. Furthermore, the lineage information (superkingdom, kingdom, phylum, class, order, family, genus, and species) for these endophytes was obtained based on their Taxonomy ID. To provide more comprehensive information, we also collected data on the host plants of endophyte, the plant parts from which these endophytes were isolated, and the geographic information of the host plants. Moreover, we collected data on the content of different NPs produced by these endophytes under various fermentation conditions, which are also available in NPASS 2.0 and NPcVar [36].

#### Biological activity and target data composition

A significant amount of biological activity data about endophytic NPs was extracted from the selected literature. The bioactivities in EMNPD were carefully categorized, including anti-bacterial activity, cytotoxic activity, and anti-inflammatory activity. The biological activity data also included target name, target type (protein, cell line, organism), target organism, potency (strong, moderate, weak, active, and inactive), activity type (e.g., IC50, MIC, EC50), activity value, control name, control activity values, and assay description. To provide authoritative information about the targets, proteins were mapped to UniProt [37] and ChEMBL, and family classification information was obtained from UniProt. Cell lines were mapped to ChEMBL, the Cell Line Ontology [38], the Experimental Factor Ontology [39], the Cellosaurus [40], and the Library of Integrated Network-based Cellular Signatures (LINCS) NIH program [41], and the classification of cell lines was relied on the categories and sampling sites in the Cellosaurus database. Lineage information for target organisms was also obtained based on the species names and was retrieved through the NCBI Taxonomy database.

#### Current database content and statistics

**Fig. 3:**
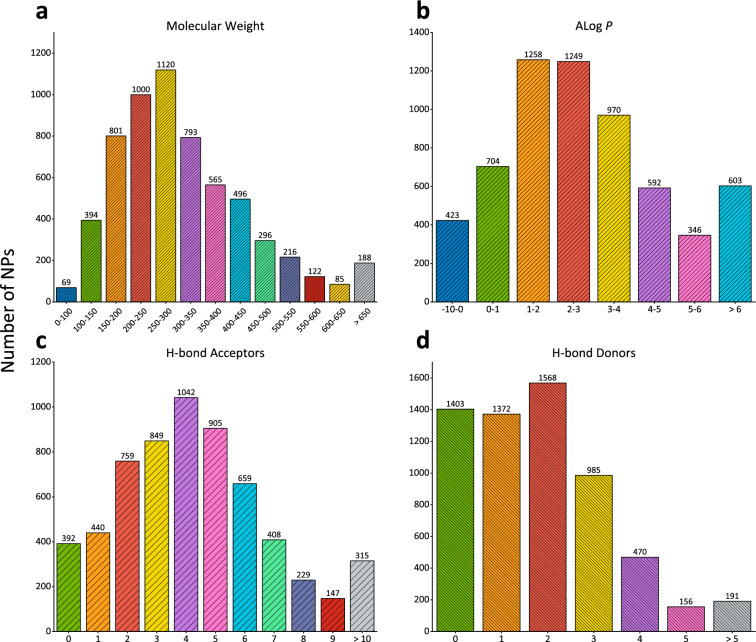
Distribution of the physio-chemical properties of compounds in EMNPD. **a** Molecular weight, **b** ALog *P*, **c** H-bond acceptors, and **d** H-bond donors

EMNPD currently contains 6632 unique NPs collected from 1017 endophytic microorganisms, which were obtained from 1016 scientific literature sources. These NPs are classified into 21 different chemical superclasses using the ClassyFire web server. The top five superclasses are ‘Organoheterocyclic compounds’ (1774), ‘Lipids and lipid-like molecules’ (1410), ‘Benzenoids’ (1036), ‘Organic oxygen compounds’ (585), and ‘Organic acids and derivatives’ (493). The distribution of MW, ALog *P*, HBA, and HBD is shown in Fig. 3. According to Lipinski’s “Rule of Five” [42], 78% (4798) of the NPs in EMNPD comply with all five rules. The endophytic microorganisms in EMNPD originate from various species sources, distributed across two kingdoms or superkingdoms (87.5% fungi, 12.5% bacteria), eight phyla, 17 classes, 47 orders, 107 families, and 192 genera. For the content data of NPs under different fermentation conditions, the current EMNPD contains 7847 records of content data for 5496 NPs generated by 816 endophytic microorganisms under 1101 various fermentation conditions. In terms of biological activity data, there are 2548 compounds (NPs with potency ranging from strong to weak and active) mapped to 1072 targets, encompassing 86 distinct bioactivities and a total of 9457 biological activity records. Furthermore, there are 2939 compounds (NPs with inactive bioactivity) mapped to 834 targets, comprising 63 bioactivities and a total of 15,095 biological activity records. These targets include 94 proteins, 282 cell lines, and 910 organisms. Protein targets are classified into 39 different protein families, such as the Tyr protein kinase family, Glycosyl hydrolase 13 family, and Ser/Thr protein kinase family. Cell line targets consist of 234 cancer cell lines, 16 transformed cell lines, 12 spontaneously immortalized cell lines, 5 finite cell lines, 3 hybrid cell lines, and several other types. Organism targets are distributed across five kingdoms or superkingdoms (49.4% bacteria, 37.5% fungi, 3.6% metazoa, 3.5% viridiplantae, and 1.5% viruses), 24 phyla, 41 classes, 87 orders, 134 families, 202 genera, and 910 species.

### Database construction and implementation

The construction of EMNPD involved the utilization of Python’s Django framework for the back-end, complemented by HTML, CSS, and JavaScript for the front-end web interface. MySQL was selected as the relational database system for efficient data storage and management. The web application was deployed on an Ubuntu Linux system, ensuring a stable and secure environment. To enhance data visualization capabilities, the JavaScript graphics library ECharts (https://echarts.apache.org/en/index.html) was incorporated, allowing for interactive and visually captivating representations of the NP data and its bioactivity information. Web access was facilitated through the Nginx web server, while uwsgi facilitated seamless interactions between Django and the proxy server, providing a scalable platform for the management, visualization, and analysis of endophyte bioactivity NPs data.

## Utility and discussion

### Web interface

EMNPD is equipped with a user-friendly online interface, providing five distinct pages: Home, Search, Browse, Download, and Help. On the Search and Browse pages, EMNPD offers powerful and diverse search functionalities along with intuitive visualizations to assist users in finding and exploring their desired content.

### Data searching

**Fig. 4:**
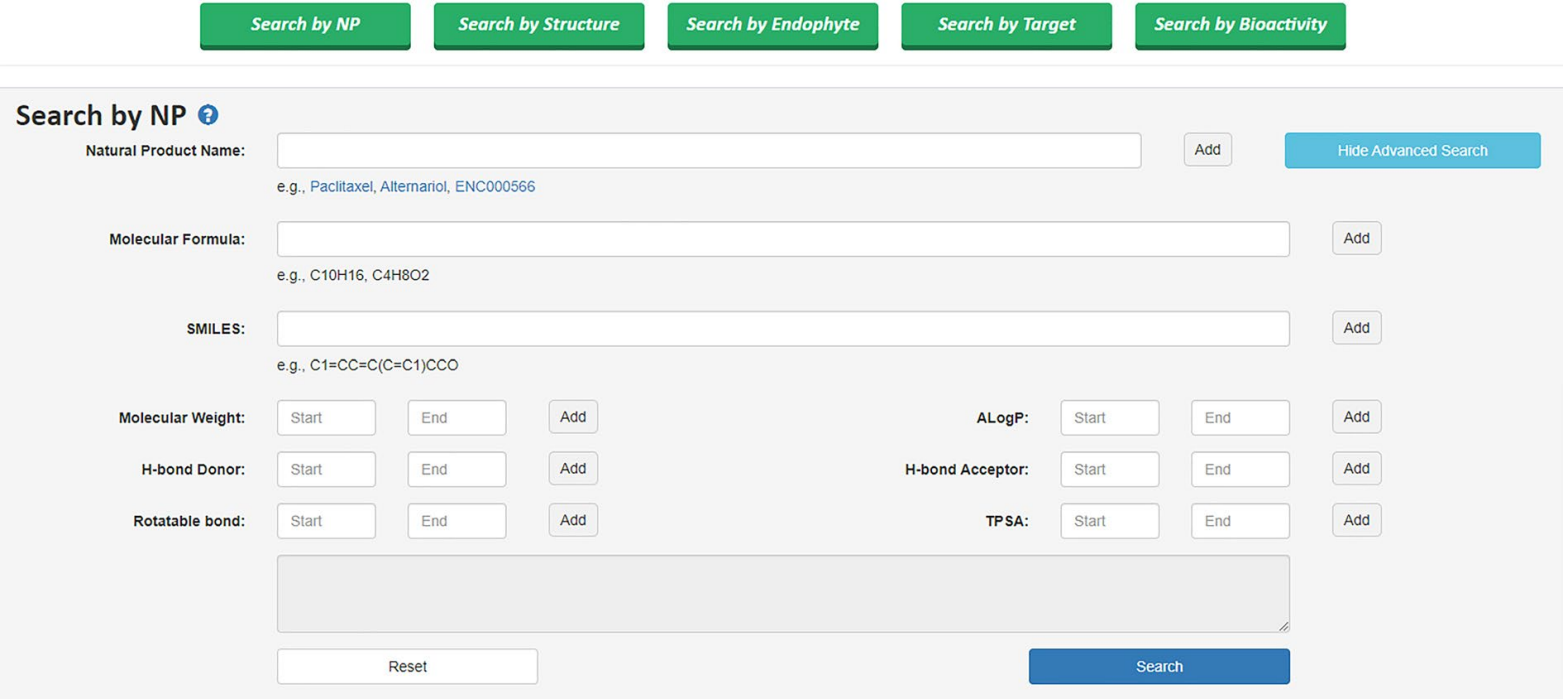
The search page of EMNPD provides multiple search options. Users can switch to the advanced search mode by clicking on “Advanced Search.” After entering the desired criteria, click the “add” button to combine search terms and create a query for searching

On the Search page of EMNPD, users can search the database content based on NPs, targets, endophytic microorganisms, and bioactivity. NP search can be performed by entering the NP name or EMNPD identifier. Additionally, the NP query page offers potent advanced search capabilities is demonstrated in Fig. 4. This allows users to specify any number of query conditions, which can be combined using boolean operators such as “AND,” “OR,” or “NOT.” The available query conditions include molecular formula, SMILES notation, log *P* range, MW, HBA, HBD, number of RB, and TPSA range. NP search can also be conducted by inputting the SMILES string in the structure input field or drawing the structure using the provided Ketcher molecular editor (Version 2.7.2) [43] in the “Search by Structure” section of the search page. Searching for endophytic microorganisms and targets (proteins, cell lines, and organisms) can be done by entering their names or EMNPD identifiers. Furthermore, EMNPD offers a dual dropdown component search method for bioactivity. The first dropdown provides options for the potency of bioactivities (Strong, Moderate, Weak, Active, Inactive), and the second dropdown consists of 91 different bioactivities, allowing users to freely combine the contents of these two dropdowns for searching.

### Data browsing

**Fig. 5:**
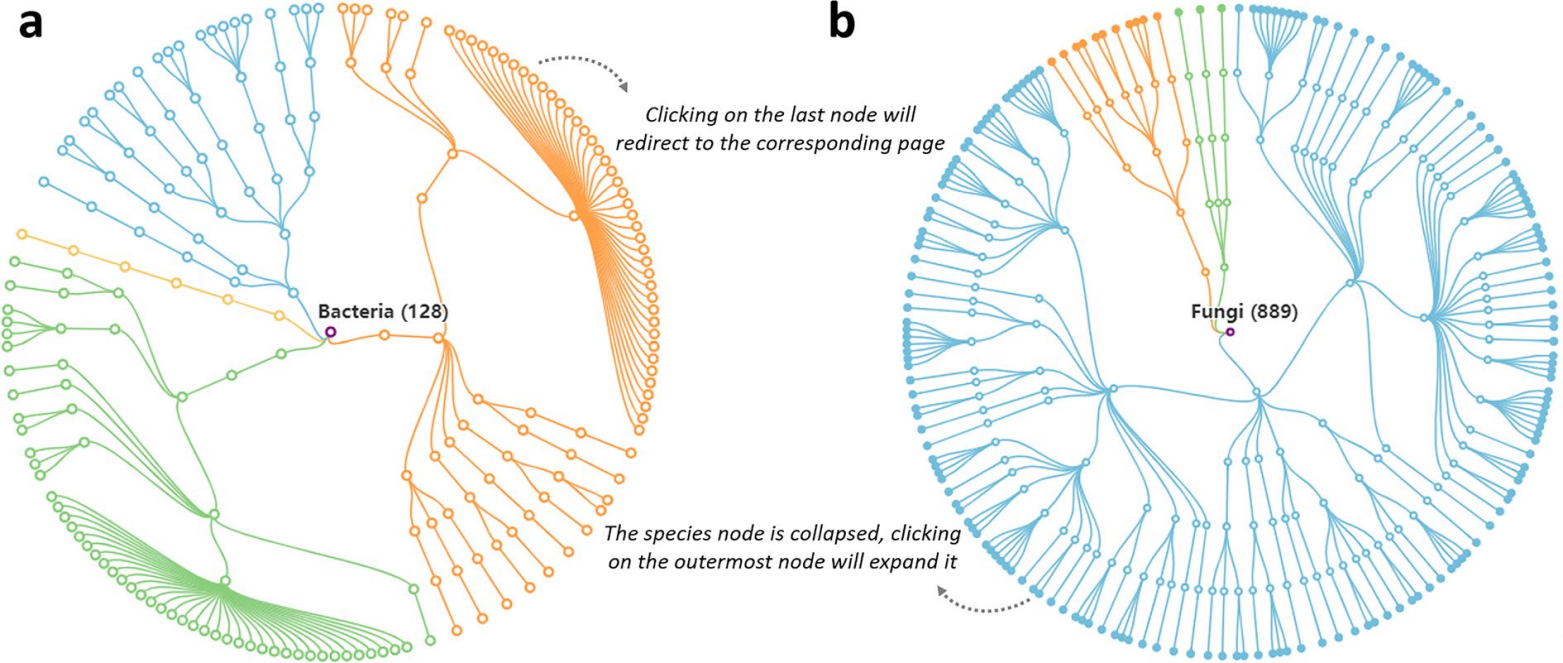
Data visualization for the taxonomy of endophytic microorganisms in EMNPD. **a** The taxonomy tree of endophytic bacteria enables users to expand specific sections of interest and explore the hierarchy to access entries at the last node. **b** The taxonomy tree of endophytic fungi collapses the final nodes representing species for enhanced visualization. Users can expand the solid nodes by clicking on them, granting access to the corresponding entries

On the Browse page, users can browse the database based on the chemical classification of NPs, the lineage of endophytic microorganisms and target species, the family classification of protein targets, and the category of cell line targets. In order to enhance user comprehension and facilitate data exploration within EMNPD, interactive data visualizations have been developed using ECharts. These visualizations include Bar chart, Tree chart, and Sunburst chart. The Bar chart visually represents the distribution ranges of MW, ALog *P*, HBA, and HBD for NPs (Fig. 3). By clicking on each bar, users can navigate to the corresponding search page with matching criteria. The Tree chart illustrates the taxonomic lineage information of endophytic microorganisms, as depicted in Fig. 5. Species differentiation is achieved by employing distinct colors based on their phyla. Each node, except for the root and leaf nodes, can be expanded or collapsed by clicking. Furthermore, the Sunburst chart displays the hierarchical structure of different classifications for target proteins and cell lines. Clicking on each section allows users to expand the chart, with the outermost nodes representing various endophytic microorganisms or targets.

### Downloads

All data displayed on the EMNPD website, including information on NPs, endophytic microorganisms, targets, and biological activity data, is integrated and available for download at the Download page. EMNPD is accessible to all users free of charge, and no login credentials are required. You can access and download all EMNPD data online via the following links: http://emnpd.idrblab.cn/download/, https://github.com/boilism/EMNPD, and https://figshare.com/articles/dataset/EMNPD_Download_Data/24078474.

## Conclusions

Endophytic microorganisms serve as a treasure trove of novel secondary metabolites, producing structurally diverse NPs with various potent biological activities. Particularly in the areas of antimicrobial and anticancer research [44, 45], they offer new avenues for drug discovery. However, these valuable resources from endophytic microorganisms have yet to be fully utilized. Despite the daily discovery of numerous novel and active endophytic microorganisms NPs, the slow pace of information updates means that it may take several years for these NPs to be included in large-scale NP databases. Therefore, information sharing is crucial for the research and development of endophytic microorganisms NPs.

To fully explore the chemical diversity of endophytic microorganisms NPs and their potential in drug discovery, we have established the first knowledge repository of endophytic microorganisms NPs, called EMNPD. The database is to provide the scientific community with comprehensive data, including a set of interactive visualization tools, to explore the chemical diversity of endophytic microorganisms NPs. EMNPD is fully searchable and downloadable, allowing researchers to query and browse data from various perspectives. In the future, with the growing interest in the study of endophytic microorganisms, we anticipate that this platform will become a valuable and comprehensive repository of endophytic microorganisms NPs, leading the way in a new wave of drug discovery.

## Data Availability

All EMNPD data can be freely downloaded at http://emnpd.idrblab.cn/download and https://figshare.com/articles/dataset/EMNPD_Download_Data/24078474. To facilitate the local deployment of the database, we have included a Docker container in the repository, which is accessible at https://github.com/boilism/EMNPD.
